# Smartphone barrier: uncovering the digital divide in mHealth prevention among disadvantaged middle-aged and older-aged UK communities

**DOI:** 10.1136/bmjdhai-2025-000069

**Published:** 2025-08-16

**Authors:** Rachael Brooks, Marieke P Hoevenaar-Blom, Nicola Coley, Eric P Moll van Charante, Linda Barnes, Edo Richard, Carol Brayne, on behalf of the PRODEMOS consortium

**Affiliations:** 1 Department of Psychiatry, University of Cambridge, Cambridge, UK; 2 Department of Public and Occupational Health, Amsterdam Public Health Research Institute, Amsterdam University Medical Center (UMC), University of Amsterdam, Amsterdam, The Netherlands; 3 Aging team, Center for Epidemiology and Research in Population Health (CERPOP), University of Toulouse-INSERM UMR1295, Toulouse, France; 4 Department of Epidemiology and Public Health, Toulouse University Hospital, Toulouse, France; 5 IHU HealthAge, Toulouse, France; 6 Department of General Practice, Amsterdam Public Health Research Institute, Amsterdam University Medical Center (UMC), University of Amsterdam, Amsterdam, The Netherlands; 7 Department of Neurology, Radboud University Donders Institute for Brain, Cognition and Behaviour, Nijmegen, The Netherlands

**Keywords:** Preventive Medicine, Public Health

## Abstract

**Objectives:**

To investigate the response to an invitation to participate in a blended mobile Health intervention among adults living in deprived areas.

**Design:**

Secondary analysis of recruitment data from a randomised controlled trial.

**Setting:**

A primary care population from disadvantaged geographies in the UK.

**Participants:**

Individuals in mid-to-late life at increased risk of developing dementia based on their risk profile.

**Interventions:**

Invitation by letter from GP, followed by a text reminder by phone.

**Main outcome measures:**

(1) Response rate to the invitation. (2) Association of response with GP practice size, area-level deprivation and COVID-19 restrictions. (3) Reasons for non-participation.

Univariate linear regression analyses were performed to assess the association between practice size and area deprivation (Index of Multiple Deprivation), with positive response rates per GP practice. The impact of COVID-19 isolation policies was assessed visually by comparing positive response rates across different restriction phases.

**Results:**

Of the 13 814 invited participants, 12 816 (93%) did not respond. Of the 998 (7%) who responded, 690 (69%) were willing to participate (positive response), while 308 (31%) declined (negative response). Of the 308 negative responders, 209 provided a reason for not being willing or able to participate. The most frequently cited reason was the requirement to use a smartphone (40%), with 33% reporting they did not have a smartphone or internet access and 7% stating that they were unable or willing to use one. There was no association between response rate and COVID-19 restrictions on public life.

**Conclusions:**

In deprived areas, most people invited to participate in a dementia prevention study via their GP practice did not respond. Smartphone use emerged as a barrier to participation in this population. Our results suggest that major efforts are needed to reduce participation bias, increase generalisability of study results and address health inequalities in preventive interventions.

**Trial registration number:**

ISRCTN15986016.

## Introduction

People with low socioeconomic position (SEP) are under-represented in research^1 2^ leading not only to reduced generalisability of findings to these groups, but also to participation and retention bias.^3–6^ This issue is particularly pronounced in intervention studies involving technology, such as mobile Health (mHealth) interventions, where access to necessary hardware and digital literacy is more limited among those with low SEP.^7^ The lack of low SEP individuals participating in research delays potential advancements in mHealth applications for people living in low SEP areas who historically experience health disparities.^8^ To improve recruitment of individuals from these groups, it is crucial to understand the recruitment process and reasons for non-participation. In the present study, we explore the response of people with low SEP to an invitation to participate in a blended mHealth intervention.^9 10^ Specifically, we assess rates of non-response, positive response and negative response (active decline), while also exploring the potential influence of external factors such as GP practice size and deprivation ranking, and COVID-19 isolation policies at the time of recruitment. We also provide an overview of individual reasons for declining participation.

## Methods

The ‘PRevention Of DEmentia using MObile phone applicationS (PRODEMOS)’ trial (Register No.: ISRCTN15986016) was a prospective, randomised, open-label blinded endpoint (PROBE) clinical trial with 12–18 months intervention and follow-up in the UK and China.^9 10^ The trial investigated whether people aged 55–75 years at elevated risk of dementia from low SEP areas in the UK (defined as ≤3 on the Index of Multiple Deprivation (IMD): higher index is less deprivation)^11^ and the general population in China could benefit from a coach-supported high-level (mHealth) application,^12^ designed to reduce dementia risk factors, including hypertension, dyslipidaemia, physical inactivity and adiposity. The requirement for the IMD was based on a patient’s postcode. General practices were identified as eligible if they served those patients in deprived areas and only those meeting the postcode criteria were contacted from each surgery. Deprived areas were specifically selected due to their higher dementia risk,^13^ recognising that these populations are often underserved by preventive healthcare. Half of those eligible and agreeing to take part were randomly allocated to a blended mHealth lifestyle intervention which included access to an interactive mobile phone application and a remote health coach; the other half were allocated to a static app with basic lifestyle and health information only.^9 10^ For this paper, only data from UK participants were analysed.

Recruitment took place between January 2021 and April 2022, during which time the UK was subject to COVID-19 isolation policies of varying intensity. Identification of General Practices (primary care provision in the UK) which were research active and fell within areas of higher deprivation was conducted in conjunction with the East of England (EoE) Clinical Research Network region (CRN, a National Institutes of Health Research infrastructure across England). Potentially eligible participants were identified using primary care data—including postcode, presence of two dementia risk factors and being aged 55–75 years (see table 1 for inclusion and exclusion criteria); these people received an invitation letter from their GP via post, encouraging them to contact the study team for more information or to participate. Ten days later, a reminder text message was sent with similar content.^9 10^

**Table 1:** Inclusion/exclusion criteria

Inclusion	Exclusion
Lives in an area of socioeconomic deprivation—defined as <4 on the Index of Multiple Deprivation Age 55–75 years Two of the following dementia risk factors: High BP Overweight High cholesterol Current smoker Physically inactive History of CVD Diabetes History of depression Smartphone possession	Diagnosis of dementia Mini-mental state examination (MMSE) <24 or <21 if low education level (ISCED 1) Terminal conditions Smartphone illiteracy Severe visual impairment Participating in another randomised controlled trial on behaviour change Severe alcohol or drug abuse Language barrier*

*Participants had to be confident English speakers.

Not contacting the study team is, in this manuscript, referred to as ‘non-response’. Contacting the study team to indicate not being able or willing to participate (including perceived ineligibility) is referred to as ‘negative response’. Progressing to the screening phase is referred to as ‘positive response’. Response rates were calculated by dividing the number of non-responders, negative-responders and positive-responders by the total number of invited people.

Univariate linear regression analyses were performed using RStudio V.4.2.1 to assess the association between practice size (per 1000 registered patients) and area deprivation (IMD), with positive response rates per GP practice. The impact of COVID-19 isolation policies on positive response rates was assessed visually by comparing response rates across different restriction phases.

For those who responded negatively, reasons for not being able or willing to participate were assessed via the telephone and recorded in an open-ended format by the assessors. If multiple reasons were provided, participants were asked to identify their primary reason. Reported reasons were coded and thematically grouped by the research team.

## Results

**Figure 1:**
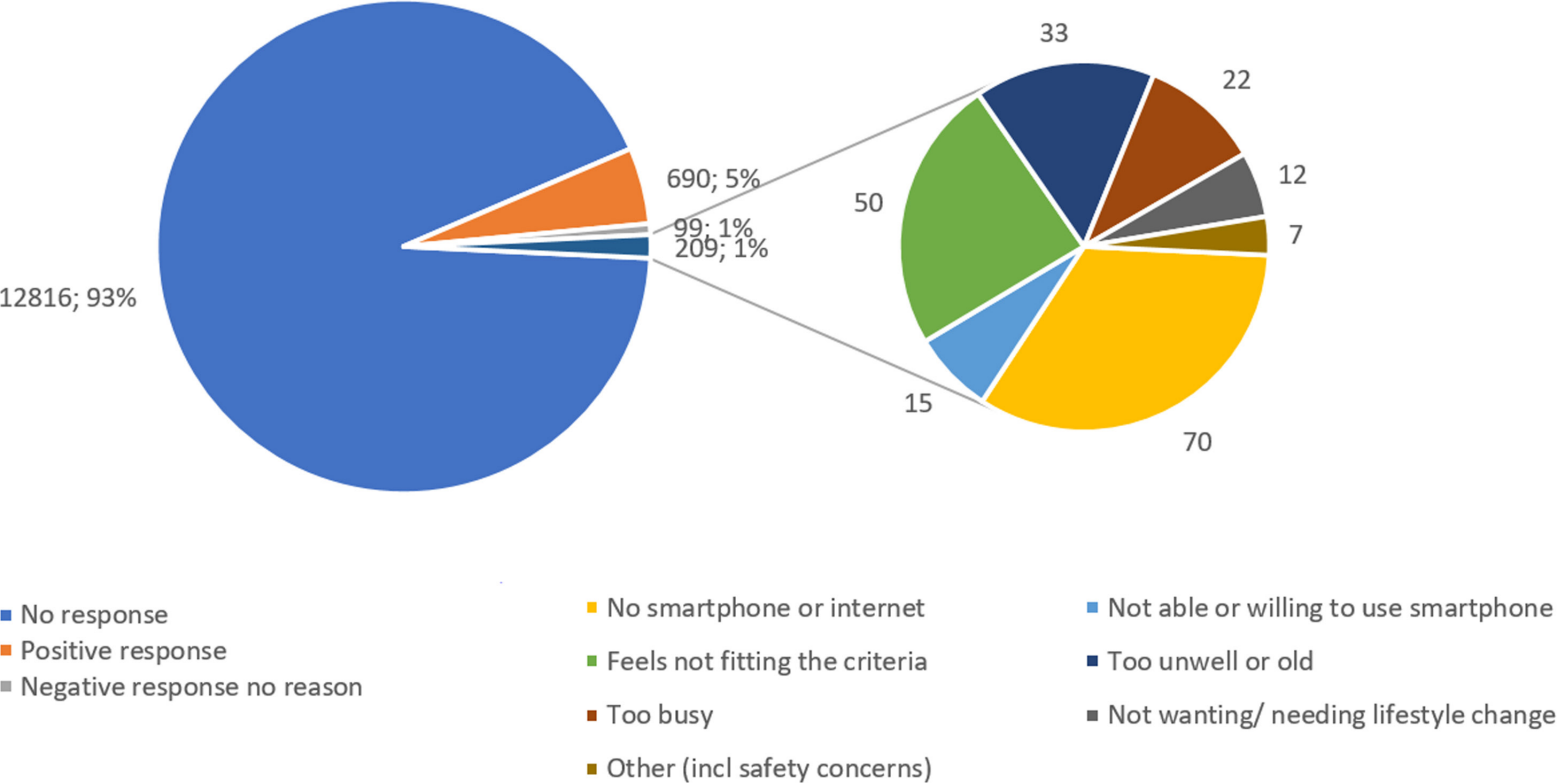
Response rates and reasons for negative response to a postal invitation (with text message reminder) to participate in the PRODEMOS study (N=13 814). PRODEMOS, PRevention Of DEmentia using MObile phone applicationS.

Of the 13 814 invited individuals, 12 816 (93%) did not respond. Of the 998 (7%) who responded, 690 (69%) responded positively and 308 (31%) negatively (figure 1). Initial (positive and negative) response to paper invitation letters was 2%, increasing to 7% after a text reminder to the participant’s mobile. Response rates (positive and negative responses combined) varied between recruiting sites from 2% to 15% (online supplemental table S1).

10.1136/bmjdhai-2025-000069.supp1 Supplementary data

There were no significant associations between IMD of the GP practice area or number of registered patients per GP practice, though trends were identified. For each 10% increment in IMD of the GP practice area, the positive response rate was 0.6 percentage points higher (95% CI: −0.2 to 1.4) and the overall response rate was 0.8 percentage points higher (95% CI: −0.3 to 1.8) (online supplemental figure S1). For every additional 1000 registered patients in a GP practice, the positive response rate decreased by 0.2% (95% CI: −0.5% to 0.1%), and the overall response rate decreased by 0.2% (95% CI: −0.6% to 0.2%) (online supplemental figure S2). Based on visual comparison, there was no apparent association between COVID-19 restrictions on public life and participation rates in the PRODEMOS study (online supplemental figure S3).

Of the 308 negative responders, 209 provided a reason for not being willing or able to participate (figure 1). The most common reason was the requirement to use a smartphone (40%), with 33% reporting they did not have a smartphone or internet access and 7% stating that they were unable or willing to use one. Other reasons included perceived ineligibility based on the inclusion criteria (24%), being too unwell or too old (16%), being too busy (11%) and not wanting or needing lifestyle changes (6%).

## Discussion

Our results provide empirical evidence of the limited reach of people from low SEP areas in the UK in the context of recruitment to a smartphone-based dementia prevention intervention. Among the small number of individuals who provided a reason for non-participation, 40% reported being unable to use or access the necessary technology. Responses were consistently very low and appeared unaffected by different phases of COVID-19 restrictions. Responses were slightly higher in areas with less deprivation and in smaller GP practices.

Recruitment took place between January 2021 and April 2022; therefore, response rates may have been impacted by the COVID-19 pandemic and accompanying restrictions.^14^ However, while the precise influence of the pandemic on recruitment remains unknown, response rates appeared unaffected by specific COVID-19 isolation policies during the recruitment phase.

The overall positive response rate of 5% was lower than that reported in similar intervention studies, where positive response rates range from 6% to 35%. However, these studies did not specifically target individuals from low SEP backgrounds^15–17^ or people of middle and older age. Our very low response rate suggests that people from socioeconomically deprived populations in the UK are unlikely to respond to invitations for a prevention study, even when the invitation is sent via their own GP—a strategy previously suggested to improve response rates.^18^ Another explanation is the recruitment method itself: ‘traditional paper invitations—despite being designed to meet ethical consent requirements—generated little interest in our study. Text message reminders on the phone increased the total response rate from 2% to 7% (data only available for total response rate, not for positive response rate). This is consistent with a Cochrane review that observed greater response with telephone reminders compared with paper reminders,^19^ and also the NHS Health Check, where uptake was higher with telephone invitations compared with paper invitations.^20^ For future studies in low SEP areas, recruitment may be enhanced by creating true community partnerships, by hiring local recruiters that can be seen as a peer to encourage participant trust,^8^ or by using additional video recruitment materials. It would be highly informative to study the difference in characteristics between the responders and non-responders or capture individual demographic characteristics—such as age—into account in the regression analyses. The design of the study and its ethical approval did not enable such capture of data. However, individual level data of the invited people who did not participate are unavailable due to collection only being permitted once informed consent was provided. Our results are descriptive and explanatory in nature. Only 1.5% of all non-participants provided a reason; those who did are unlikely to be representative of all non-responders, ironically introducing a further non-response bias. However, their responses offer valuable insights into the extremely low engagement rates of this low SEP population.^21^ A substantial proportion of those who gave a reason for not participating found the mHealth intervention inaccessible due to not owning a mobile phone with internet access or lacking the necessary digital skills—an issue previously identified in other digital interventions.^7^ This is consistent with a recent review incorporating 42 studies which concluded that important barriers for people with a low SEP were technical difficulties like lack of internet access, problems with telephones and poor signal as well as limited digital skills of users.^22^ This lack of access to technology is particularly relevant given the increasing reliance on digital health interventions and the optimism surrounding their potential by healthcare authorities. The implications extend beyond research and directly impact preventive healthcare. With an increasing shift towards digital solutions in health research, those who lack confidence in using technology or simply do not have access to it risk being excluded, further exacerbating health inequalities.^23^ ‘Providing the possibility to rent a smartphone at low or no cost could arguably increase response rates; however, such an approach may require further support.^24^ To improve engagement with underserved populations, it is crucial to develop novel methods for capturing reasons for non-participation among individuals from low-SEP backgrounds such as qualitative approaches, where there are community champions who might be able to help understand what the reasons for lack of engagement in such processes are^25 26^ or via social media.^27^ Without such insights, trial designs cannot be adapted to enhance participation and improve the generalisability of results.

To prevent widening health inequalities, recruitment strategies should specifically endeavour to reach and engage those of low SEP.^7 15 23^ Our key point is that a blanket belief that such approaches can be mass distributed with an expectation of impact is unfounded, based on our evidence presented. Future mHealth studies and practitioners trying to reach those of low SEP are recommended to make use of simple reminders, or engage with trusted community networks or key members.^28^ Providing non-digital alternatives or at least support for digital engagement by offering training, technical support or even materials like a smartphone may increase the reach towards low SEP populations.^23 24^ This will allow for better representation of people from low SEP areas in trials, improving generalisability of the results and reducing participation and retention bias and ultimately helping to minimise inequalities in access to health interventions.

## Data Availability

Data are available upon reasonable request. Please contact m.p.hoevenaarblom@amsterdamumc.nl for data request.
