# Supplementary material for: Smartphone barrier: uncovering the digital divide in mHealth prevention among disadvantaged middle-aged and older-aged UK communities
Source: BMJ Digit Health Ai. 2025 Aug 16;1(1):e000069. doi: 10.1136/bmjdhai-2025-000069 (PMC13528400; doi:10.1136/bmjdhai-2025-000069)
Supplement: Supplementary file 1 [file bmjdhai-1-1-bmjdhai-2025-000069-s001.pdf]

Supplementary materials

Title: The smartphone barrier: uncovering the digital divide in mHealth prevention among disadvantaged middle- and older-aged UK communities

Subtitle: A secondary analysis of recruitment data of a randomised controlled trial

Brooks R, *et al. BMJ Digit Health* 2025; 1:e000069. doi: 10.1136/bmjdhai-2025-000069

Table S1: Positive, negative, and non-response rates by general practice

General Practice	Letters sent	Positive Response (PR)	PR %	Negative response inc. ineligible (N)*	N %	No Response (NR)	NR%
A	280	22	7.9	8	2.9	250	89.3
B	236	16	6.8	11	4.7	209	88.6
C	2,546	98	3.9	48	1.9	2,400	94.3
D	118	3	2.5	2	1.7	113	95.8
E	301	11	3.6	5	1.7	285	94.7
F	357	12	3.4	8	2.2	337	94.4
G	522	40	7.7	19	3.6	463	88.7
H	1,271	81	6.4	30	2.4	1,160	91.3
I	440	33	7.5	19	4.3	388	88.2
J	806	79	9.8	21	2.6	706	87.6
K	980	57	5.8	14	1.4	909	92.8
L	271	20	7.4	9	3.3	242	89.3
M	303	36	11.9	9	3.0	258	85.1
N	606	14	2.3	7	1.2	585	96.5
O	699	10	1.4	6	0.9	683	97.7
Q	803	34	4.2	18	2.2	751	93.5
R	141	7	5.0	3	2.1	131	92.9
S	684	26	3.8	12	1.8	646	94.4
T	2450	91	3.7	61	2.5	2,298	93.8
All Practices	13,814	690	5.0	308	2.2	12,816	92.8

Legend: Letters represent individual recruiting practices. *The total number of participants who either actively declined participation or were found to be ineligible after responding.

Brooks R, *et al. BMJ Digit Health* 2025; 1:e000069. doi: 10.1136/bmjdhai-2025-000069

Figure S1: Positive response rates by General Practice area deprivation level

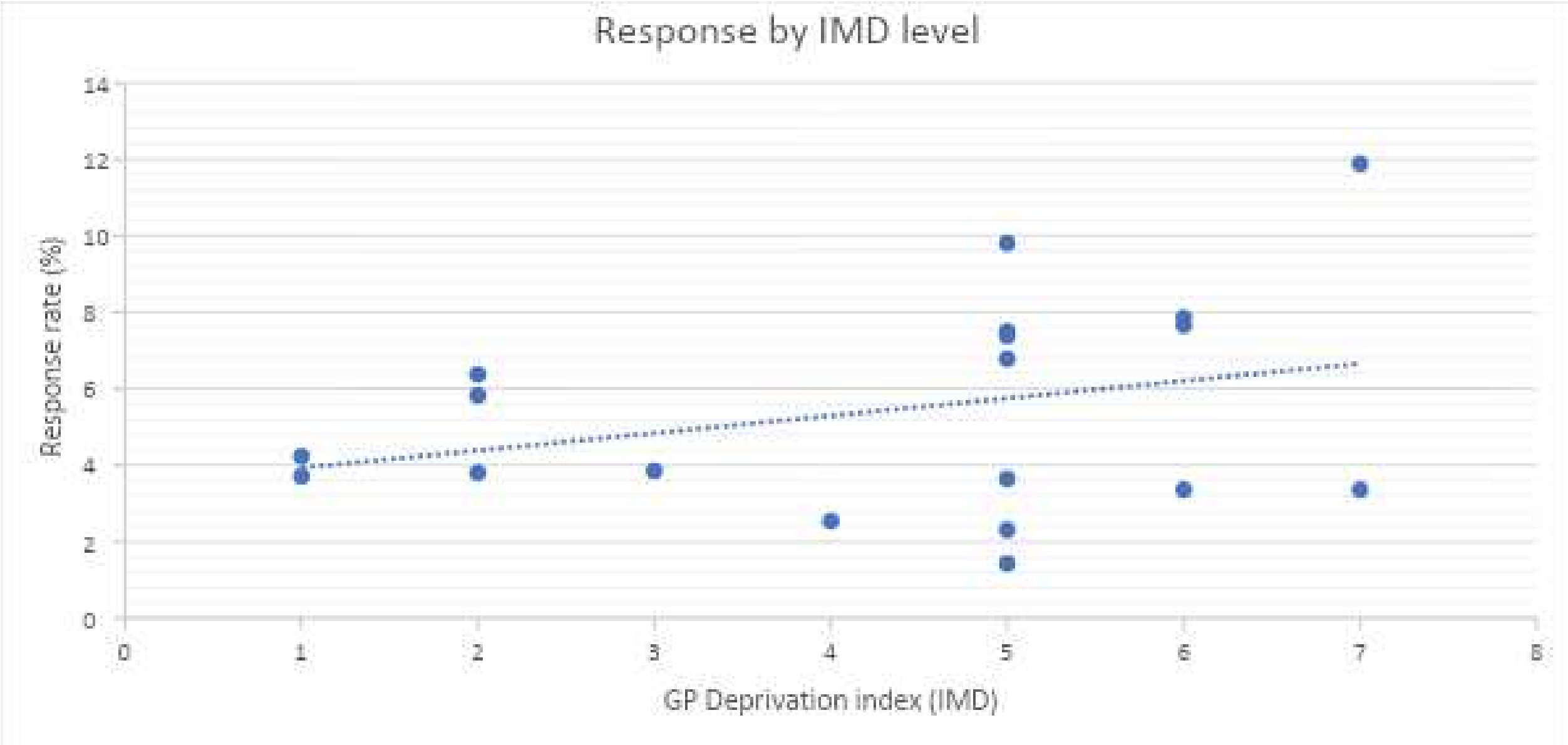

Figure S2: Positive response rates by size of general practice

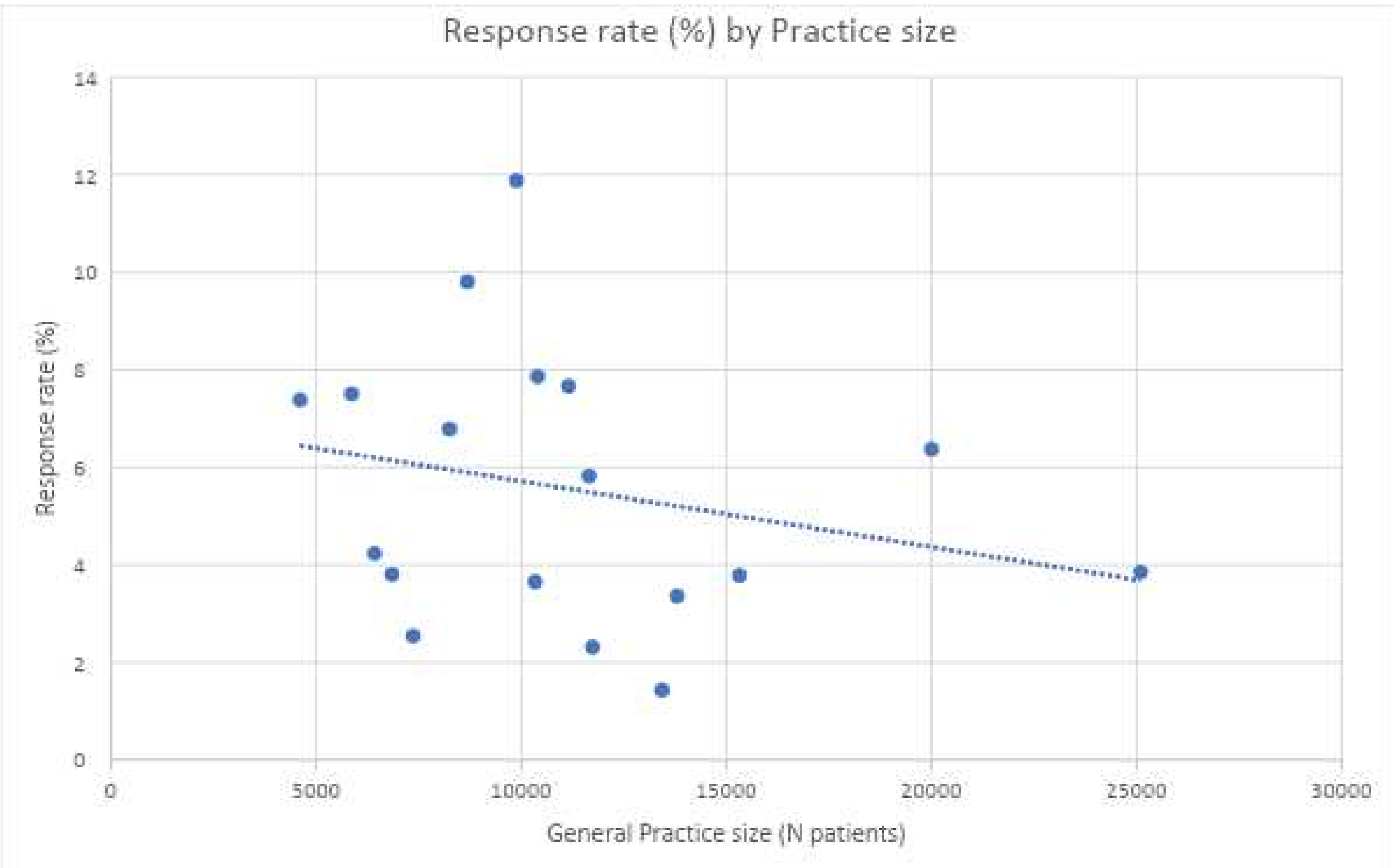

Figure S3: Response rates in individual GP practices (y-axis) in relation to COVID restrictions

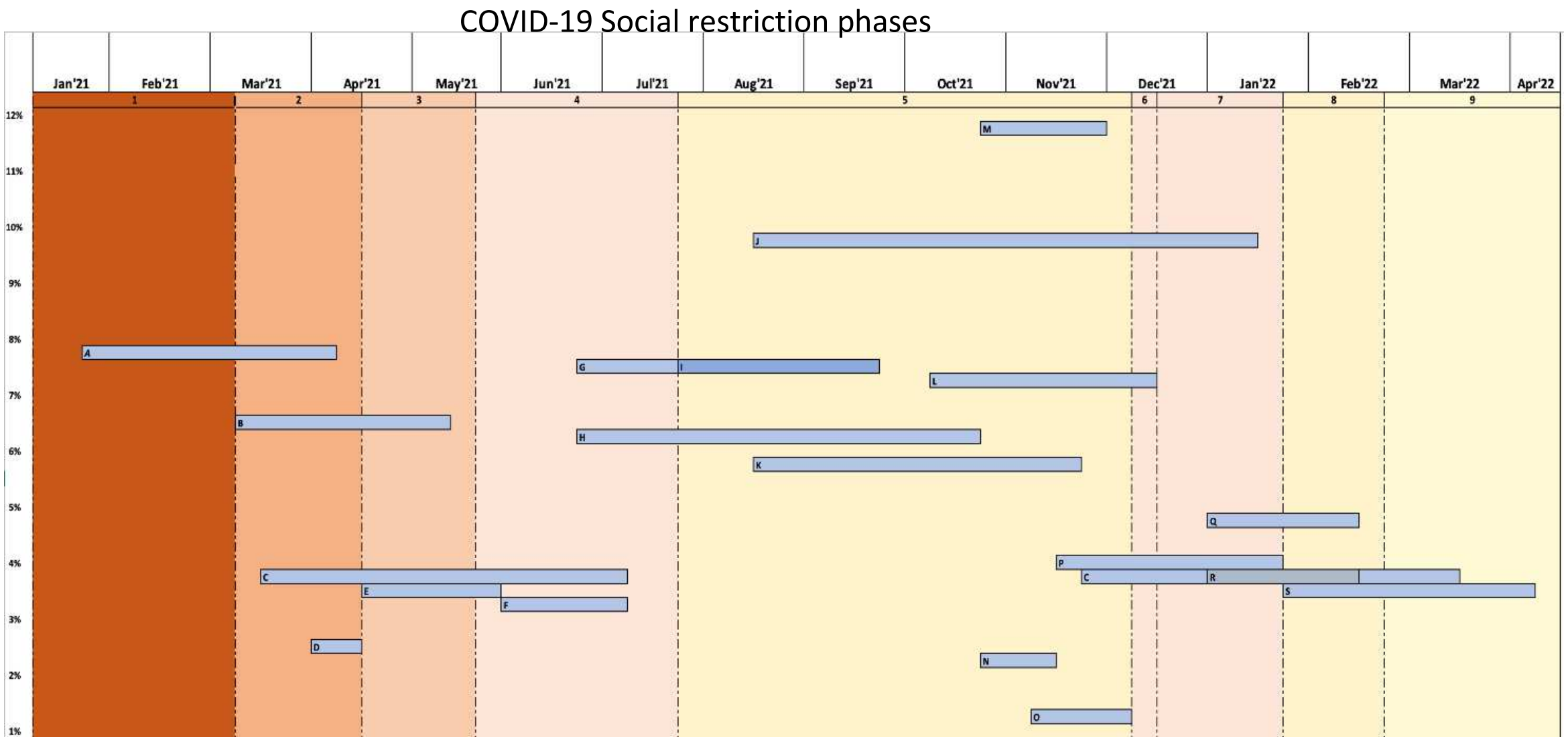

Legend: Columns represent the different phases of COVID restrictions (details of each phase are given in the table below). Blue horizontal bars represent the recruitment periods of each of the 19 general practices (corresponding to Table S1 and labelled from A to S). Columns are color coded from red (severe restrictions) to light yellow (no restrictions.

COVID19 restriction phases (x-axis)

**Stage**	**Description**	**Date started**
Stage 1	3RD National lockdown	04/01/2021
Stage 2	Schools reopen	08 /03/2021
Stage 3	Non-essential retail and outdoor hospitality allowed to re-open	12/04/2021
Stage 4	Outdoor gatherings of up to 30 people permitted, indoor gatherings of up to 6 people	17/05/2021
Stage 5	Most remaining restrictions lifted	19/07/2021
Stage 6	Move to plan B – mandatory face masks in most public indoor venues	08/12/2021
Stage 7	COVID Pass introduced – required for entry into certain venues such as nightclubs	15/12/2021
Stage 8	Removal of requirement to present COVID passes at venues and events, face coverings on public transport and indoor locations, as well as the guidance to work remotely.	18/01/2022
Stage 9	Remaining COVID rules scrapped in England	24/02/2022
